# North American terrestrial CO_2_ uptake largely offset by CH_4_ and N_2_O emissions: toward a full accounting of the greenhouse gas budget

**DOI:** 10.1007/s10584-014-1072-9

**Published:** 2014-03-14

**Authors:** Hanqin Tian, Guangsheng Chen, Chaoqun Lu, Xiaofeng Xu, Daniel J. Hayes, Wei Ren, Shufen Pan, Deborah N. Huntzinger, Steven C. Wofsy

**Affiliations:** 1 International Center for Climate and Global Change Research and School of Forestry and Wildlife Sciences, Auburn University, Auburn, AL 36849 USA; 2 Environmental Sciences Division, Oak Ridge National Laboratory, Oak Ridge, TN 37831 USA; 3 School of Earth Sciences and Environmental Sustainability, North Arizona University, Flagstaff, AZ 86011 USA; 4 Department of Earth and Planetary Science, Harvard University, 29 Oxford St., Cambridge, MA 02138 USA

## Abstract

The terrestrial ecosystems of North America have been identified as a sink of atmospheric CO_2_ though there is no consensus on the magnitude. However, the emissions of non-CO_2_ greenhouse gases (CH_4_ and N_2_O) may offset or even overturn the climate cooling effect induced by the CO_2_ sink. Using a coupled biogeochemical model, in this study, we have estimated the combined global warming potentials (GWP) of CO_2_, CH_4_ and N_2_O fluxes in North American terrestrial ecosystems and quantified the relative contributions of environmental factors to the GWP changes during 1979–2010. The uncertainty range for contemporary global warming potential has been quantified by synthesizing the existing estimates from inventory, forward modeling, and inverse modeling approaches. Our “best estimate” of net GWP for CO_2_, CH_4_ and N_2_O fluxes was −0.50 ± 0.27 Pg CO_2_ eq/year (1 Pg = 10^15^ g) in North American terrestrial ecosystems during 2001–2010. The emissions of CH_4_ and N_2_O from terrestrial ecosystems had offset about two thirds (73 %±14 %) of the land CO_2_ sink in the North American continent, showing large differences across the three countries, with offset ratios of 57 % ± 8 % in US, 83 % ± 17 % in Canada and 329 % ± 119 % in Mexico. Climate change and elevated tropospheric ozone concentration have contributed the most to GWP increase, while elevated atmospheric CO_2_ concentration have contributed the most to GWP reduction. Extreme drought events over certain periods could result in a positive GWP. By integrating the existing estimates, we have found a wide range of uncertainty for the combined GWP. From both climate change science and policy perspectives, it is necessary to integrate ground and satellite observations with models for a more accurate accounting of these three greenhouse gases in North America.

**Electronic supplementary material:**

The online version of this article (doi:10.1007/s10584-014-1072-9) contains supplementary material, which is available to authorized users.

## Introduction

Radiative forcing of climate is increasing at unprecedented rates in Earth’s atmosphere, largely due to rapid increases in the atmospheric concentrations of greenhouse gases (GHGs) such as CO_2_, CH_4_, and N_2_O (Forster et al. 2007). These three GHGs combined contribute to more than 90 % of anthropogenic climate warming (Hansen et al. 2000). Atmospheric CH_4_ and N_2_O are two potent greenhouse gases which in sum contribute to more than one quarter of the anthropogenic global warming (Forster et al. 2007; USGCRP 2009). Similar to atmospheric CO_2_, atmospheric CH_4_ and N_2_O concentrations have dramatically increased since the Industrial Revolution (Forster et al. 2007; Rigby et al. 2008). During the past decades, however, less attention has been paid to terrestrial CH_4_ and N_2_O fluxes relative to the focus on regional CO_2_ budget accounting. The global warming potential (GWP) of CH_4_ is about 25 (100-year horizon) times larger than that of CO_2_, while N_2_O is about 298 times (Forster et al. 2007). Therefore, although CH_4_ and N_2_O concentrations are relatively low in the atmosphere, they are of critical significance in contributing to climate warming. Owing to more available data from field experiments and observations and improved representations of biogeochemical processes in ecosystem models, regional CH_4_ and N_2_O fluxes have been estimated in a number of recent studies (e.g., Tian et al. 2010; Xu et al. 2010; Huang et al. 2010; Li et al. 1996; Miller et al. 2012). However, the concurrent fluxes of all these three gases have not yet been well investigated (Tian et al. 2011a). Since close linkages exist among CO_2_, CH_4_ and N_2_O fluxes and one gas flux altered by environmental forces would affect the other two, a systems approach incorporating all three GHGs would be needed to provide an accurate estimate on GWP (Lu and Tian 2013).

The fluxes of CO_2_, CH_4_, and N_2_O could be greatly influenced by multiple environmental changes, such as climate, nitrogen deposition, CO_2_ fertilization, land use, and land management practices (e.g., nitrogen fertilizer uses and irrigation). Roles of these driving forces on North American CO_2_ balance are extensively studied based on the long-term experiments and observations (e.g., FLUXNET sites), remote sensing data, and modeling synthesis activities (Xiao et al. 2011; Hayes et al. 2012; Huntzinger et al. 2012), but only a few studies have investigated their roles on the fluxes of CH_4_ and N_2_O (e.g., Tian et al. 2010, 2012b; Xu et al. 2010, 2012). Many global change factors could affect the fluxes of CH_4_ and N_2_O (Huang et al. 2010; Toet et al. 2011; Banger et al. 2012; Dijkstra et al. 2012). For instance, elevated atmospheric CO_2_ might stimulate CH_4_ emission (Dacey et al. 1994), while reducing or increasing N_2_O emission (Ineson et al. 1998; Kettunen et al. 2005); ozone (O_3_) pollution might reduce CH_4_ emission (Toet et al. 2011), while stimulating or reducing N_2_O emission (Kanerva et al. 2008). Meanwhile, interactions among multiple factors may also influence CH_4_ and N_2_O fluxes. For example, one recent study reported that nitrogen deposition and elevated atmospheric CO_2_ might interactively reduce CH_4_ emission from wetlands (Pancotto et al. 2010); another study concluded that temperature and elevated atmospheric CO_2_ interactively altered seasonal variation of CH_4_ emissions (Blankinship et al. 2010). Therefore, it is necessary to study the concurrent fluxes of CO_2_, CH_4_ and N_2_O fluxes under multi-factor global changes (Tian et al. 2011a).

Based on results and data from the North American Carbon Program (NACP) Regional Interim Synthesis (Huntzinger et al. 2012; Hayes et al. 2012), Non-CO_2_ GHG regional interim synthesis (Tian et al. 2012b) and model simulations with the Dynamic Land Ecosystem Model (DLEM), this study intends to: 1) estimate the overall GWP of CO_2_, CH_4_, and N_2_O fluxes in the terrestrial ecosystems of North America; 2) quantify the relative contributions of individual environmental factors to GWP changes during recent 32 years (1979–2010); and 3) identify gaps and uncertainties in existing estimates of the GHG balances for improving climate prediction and guiding climate change policy-making in North America.

## Methods and data

The DLEM model (Tian et al. 2010, 2011a, b) was used to simultaneously quantify the magnitudes as well as spatial and temporal patterns of CO_2_, CH_4_, and N_2_O fluxes in the terrestrial ecosystems of North America, and attribute GWP variations to different environmental factors. Additionally previous studies were synthesized to provide an estimate of the uncertainty range of contemporary GWP from GHG fluxes. Magnitudes of CO_2_, CH_4_ and N_2_O sources and sinks are defined as the vertical land-atmosphere exchanges. In this study, we only estimated these fluxes from the terrestrial biosphere, while the emissions from human activities such as fossil fuel combustion, transportation, and industrial processes etc., are excluded from the estimation.

### DLEM model description

The DLEM model is a highly integrated process-based ecosystem model that couples carbon, nutrients (i.e., nitrogen and phosphorus) and water cycles in terrestrial ecosystems for estimating the hydrological, biogeochemical fluxes and pool sizes at multiple scales from site to region/globe and with time steps ranging from day to year. Through the carbon-nutrient-water coupling, DLEM is capable of simultaneously depicting the biosphere-atmosphere exchange of CO_2_, CH_4_ and N_2_O under multiple natural and anthropogenic disturbances. DLEM has been widely applied and evaluated to estimate CO_2_, CH_4_ and N_2_O fluxes at multiple sites and regions including China (Ren et al. 2011, 2012; Tian et al. 2011a,b; Lu et al. 2012; Xu and Tian, 2012; Lu and Tian 2013), the southern US (Tian et al. 2012a; Chen et al. 2012, 2013; Zhang et al. 2012), North America (Tian et al. 2010, 2012b; Xu et al. 2010, 2012; Huntzinger et al. 2012), and the global land ecosystem (Melton et al. 2013; Tian et al. 2013). In the Supplementary Material, we briefly present the key processes involved in simulating land-atmosphere exchanges of CO_2_, CH_4_ and N_2_O in DLEM. Additional details about the DLEM model can be found in our previous publications (e.g., Tian et al. 2010, 2011a, b; Liu et al. 2013).

### Model input data

The gridded input data sets at a spatial resolution of 32 km × 32 km including climate (temperature, precipitation, humidity, and solar radiation), tropospheric ozone (O_3_) level, atmospheric CO_2_ concentration and nitrogen deposition rate, land use and cropland management practices (i.e., fertilization and irrigation) were generated for driving the DLEM model simulation in North America (including the United States, Canada and Mexico) during 1979–2010 (Tian et al. 2010). Methods of these data generation is described in the Supplementary Material. Additional descriptions of changes in environmental driving factors were also presented in Tian et al. (2010) and Xu et al. (2010, 2012)

### Model simulations and evaluation

For this study, several model experiments were designed to address the effects of individual and combined environmental factors on CO_2_, CH_4_, and N_2_O dynamics. Seven transient experiments include: 1) Combined: changes of all environmental factors are considered; 2) CLM: climate variability alone; 3) LC: land cover change alone; 4) NDEP: nitrogen deposition change alone; 5) Nfer: nitrogen fertilizer use change alone; 6) O_3_: tropospheric O_3_ change alone; 7) CO_2_: atmospheric CO_2_ concentration change alone. We aim at investigating the CO_2_, CH_4_, and N_2_O fluxes during 1979–2010, but we start model simulations from 1900 to consider the legacy effects before 1979. Seven additional baseline experiments were conducted to remove system errors. The baseline experiments use the transient environmental data during 1900–1978 and the input drivers remain constant after 1978. The simulated CO_2_, CH_4_ and N_2_O fluxes under different experiments are the differences between transient and corresponding baseline experiments. The interactive effects among multiple environmental factors were calculated as: Interaction = Combined - CLM - LC - NDEP - Nfer - O_3_ – CO_2_.

The DLEM model performance for simulating CO_2_, CH_4_ and N_2_O fluxes were widely evaluated against field observational/experimental data (e.g., the eddy flux towers, the Long Term Ecological Research Network, and other independent sites), other modeling results (e.g., inverse and forward modeling), and regional inventory data (e.g., Forest Inventory and Analysis). The evaluation results for CO_2_ fluxes were shown in Chen et al. (2012), Schwalm et al. (2010), Tian et al. (2011a, b, 2012a, c) and Lu et al. (2012), while evaluations for CH_4_ and N_2_O fluxes were shown in Tian et al. (2010, 2011b, 2012c, 2013), Ren et al. (2011), Lu and Tian (2013) and Xu et al. (2010, 2012). The evaluation results indicated that the DLEM model is able to capture the monthly/seasonal variations in CO_2_, CH_4_ and N_2_O fluxes at a relatively high confidence level. The comparisons between model-simulated and field observed factorial contributions to these GHGs were also shown in the above literature. The DLEM model parameterization and implementation are described in the Supplementary Material.

### Integration of previous studies and DLEM simulation results

To estimate the uncertainty range, we synthesized many previous estimates on these three GHGs, including inventory data, as well as forward and inverse modeling results for the terrestrial ecosystems in North America. The study region covers the three countries of North America (Canada, USA, and Mexico) and the reference time period was approximately 2001–2010 for contemporary analysis and 1979–2010 for historical change analysis. The data sources mainly include: (1) the contemporary estimate of CO_2_ fluxes derived from the NACP- Regional and Continental Interim Synthesis studies that integrated the forward modeling results, inverse modeling results and inventory data at 97 reporting zones (Hayes et al. 2012; Huntzinger et al. 2012), which cover the majority of US states, Canadian managed ecoregions, and Mexican states for which inventory data were available; (2) the contemporary estimate of CH_4_ and N_2_O budgets in the North American terrestrial ecosystems, derived from a recent synthesis (Tian et al. 2012b); and (3) the gridded, time-series data set of DLEM-simulated CH_4_ and N_2_O fluxes, as well as the contributions of multiple environmental factors (Tian et al. 2010, 2012b; Xu et al. 2010, 2012). In this study, we combined the synthesized CO_2_ flux estimates and DLEM-simulated CH_4_ and N_2_O fluxes to examine net GWP for these 97 reporting zones. The multi-approach estimations during the 2000s are used to generate a “best-estimate” of the contemporary CO_2_, CH_4_ and N_2_O fluxes, while the DLEM simulation results are used to identify the contributions of different environmental factors on GHG fluxes for the period 1979–2010. Due to relatively slow changes in environmental factors, a long-term period is better to represent their effects on GWP.

The uncertainty ranges of the estimated CO_2_, CH_4_ and N_2_O fluxes, as well as the combined GWP, are expressed as the mean value ± 2 standard errors. An error propagation method is used when we combine the standard errors from multiple sources without original data (e.g., the integration of standard errors from forward and inverse modeling methods).

### Calculation of GWP

The GWP of a GHG is defined as the ratio of the time-integrated radiative forcing from the instantaneous release of 1 kg of a trace substance relative to that of 1 kg of a reference gas. Negative GWP indicates GHG uptake from the atmosphere and a potential climate cooling effect while positive GWP indicates GHG release to the atmosphere and a potential climate warming effect. The equation to calculate GWP is:

1 $$ GWP={F}_{CO2-C}\times \frac{44}{12}+{F}_{CH4-C}\times \frac{16}{12}\times R{F}_{CH4}+{F}_{N2O-N}\times \frac{44}{28}\times R{F}_{N2O} $$

Where *F*_*CO*2 − *C*_, *F*_*CH*4 − *C*_ and *F*_*N*2*O* − *N*_ are annual fluxes of CO_2_, CH_4_ and N_2_O between terrestrial ecosystems and the atmosphere based on mass of C and N, respectively. The fractions of 44/12, 16/12 and 44/28 convert C and N mass into mass of CO_2_, CH_4_ and N_2_O. *RF*_*CH*4_  and *RF*_*N*2*O*_  are constants indicating radiative forcing of CH_4_ and N_2_O in terms of a CO_2_ equivalent unit, and were assigned to 25 and 298, respectively at 100 year time horizon (Forster et al. 2007).

## Results and discussion

### Contemporary GWP estimates for terrestrial North America

**Fig. 1:**
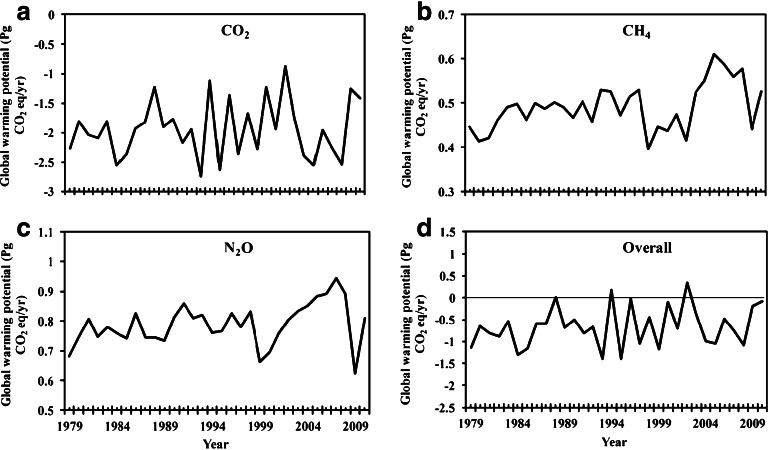
Interannual variations of global warming potential (Pg CO_2_ eq/year) for CO_2_ (**a**), CH_4_ (**b**), N_2_O (**c**) and their overall fluxes (**c**) during 1979–2010. *Note* Positive values indicate a potential net contribution to climate warming; the data was based on our previous publications (i.e., Tian et al. 2010; Xu et al. 2010, 2012)

Based on DLEM simulated CO_2_, CH_4_ and N_2_O fluxes, our “best estimate” of the overall GWP in North American terrestrial ecosystems during 2001–2010 was −0.50 ± 0.27 (mean ± 2 SE) Pg CO_2_ eq/year (1 Pg = 10^15^ g), ranging from −1.08 Pg CO_2_ eq/year in 2008 to 0.34 Pg CO_2_ eq/year in 2002 (Table 1; Fig. 1). The overall GWP was calculated from net balance of three gases: CO_2_ (−1.83 ± 0.34 Pg CO_2_ eq/year), CH_4_ (0.52 ± 0.04 Pg CO_2_ eq/year), and N_2_O (0.82 ± 0.06 Pg CO_2_ eq/year). The terrestrial CO_2_ sink greatly reduced atmospheric radiative forcing; however, the emissions of CH_4_ and N_2_O could largely offset this cooling effect by a range of 58 % ~ 138 % (with a mean of 73 % ± 14 %; Table 1). The overall GWP had a wide range, yet it indicated that North American terrestrial ecosystems were generally contributing to climate cooling during the recent decade.

**Table 1:** CO_2_, CH_4_ and N_2_O fluxes and their overall global warming potential (Pg CO_2_ eq/year) in the 2000s as estimated by the DLEM model

Countries	CO_2_	CH_4_	N_2_O	Overall GWP	Offset rate by CH_4_ and N_2_O
US	−1.24 ± 0.41	0.23 ± 0.04	0.48 ± 0.06	−0.53 ± 0.38	57 % ± 8 %
Canada	−0.54 ± 0.27	0.30 ± 0.07	0.15 ± 0.02	−0.09 ± 0.21	83 % ± 17 %
Mexico	−0.05 ± 0.23	−0.007 ± 0.001	0.19 ± 0.04	0.13 ± 0.21	329 % ± 119 %
North America	−1.83 ± 0.34	0.52 ± 0.04	0.82 ± 0.06	−0.50 ± 0.27	73 % ± 14 %

### Temporal patterns of GWP and contributions from multiple environmental factors

Due to the lack of other available data for simultaneously estimating CO_2_, CH_4_, and N_2_O fluxes over a long-term period, we used estimates from the DLEM model alone to analyze temporal patterns of GWP and contributions of multiple environmental factors during 1979–2010. Net GWP was negative during most years due to large carbon uptake in North America, with the largest negative values in 1993 (−1.39 Pg CO_2_ eq/year) and 1995 (−1.39 Pg CO_2_ eq/year); however, the positive GWP occurred in some years with extreme drought events, such as 1988 (0.02 Pg CO_2_ eq/year), 1994 (0.17 Pg CO_2_ eq/year), and 2002 (0.34 Pg CO_2_ eq/year) (Fig. 1). The GWP values in these three extreme dry years were significantly (*P* < 0.01) higher than those in normal years. It is notable that CH_4_ and N_2_O emissions offset the CO_2_ sink by 115 % and 138 % during 1994 and 2002, respectively, indicating that the terrestrial ecosystems in North America might act as a significant contributor to global warming due to extreme drought events.

**Fig. 2:**
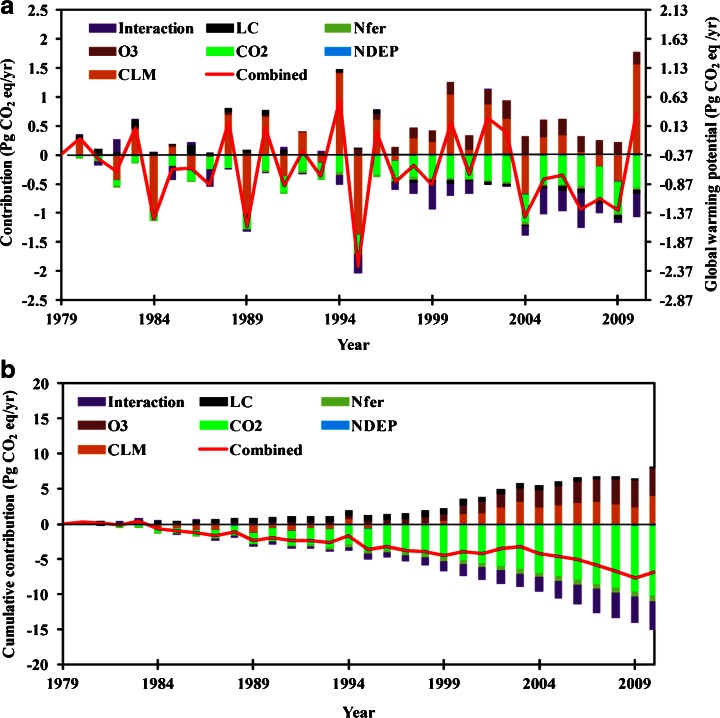
Annual (**a**) and cumulative (**b**) contributions of different environmental factors to changes in global warming potential (Pg CO_2_ eq/year) in the terrestrial ecosystems of North America. The secondary Y-axis in (**a**) is the annual actual global warming potential under combined scenario (contribution of individual factors + baseline); *Combined* combined scenario (include all the environmental factors), *LC* land conversion, *Nfer* N fertilizer use, *O*_*3*_ O_3_ pollution, *CO*_*2*_ atmospheric CO_2_, *NDEP* N deposition, *CLM* climate variability, *Interaction* interaction among multiple factors calculated as: *Combined* – *LC* – *Nfer* – *O*_*3*_ – *CO*_*2*_ – *NDEP* – *CLM*

During 1979–2010, we found that interannual variation in overall GWP was largely determined by the fluxes of CO_2_, while CH_4_ and N_2_O emissions showed smaller variation. To identify the possible causes of GWP variation in North American terrestrial ecosystems, we conducted several factorial simulation experiments using the DLEM model. During 1979–2010, the interannual variation of the overall GWP was primarily determined by climate variability (R^2^ = 0.82; *P* < 0.05; Fig. 2a). Climate change increased GWP by 3.99 Pg CO_2_ eq in total during the study period, implying a positive feedback between climate change and global warming. Land use change increased GWP before 1997 and then reduced it after that, with a total increase of GWP by 0.23 Pg CO_2_ eq during the entire study period (Fig. 2b). Elevated atmospheric CO_2_ concentration was the largest contributor to the reduction of GWP (reduced GWP by 9.92 Pg CO_2_ eq) in North America, which resulted in a dilemma that increased atmospheric CO_2_ could directly lead to global warming while indirectly mitigating the warming trend through stimulating plant growth and carbon uptake. Increasing nitrogen deposition slightly reduced GWP by 0.20 Pg CO_2_ eq during 1979–2010. Although elevated nitrogen deposition resulted in a small CO_2_ sink (0.41 Pg CO_2_ eq), it caused an increase in N_2_O emission (Xu et al. 2012). Likewise, nitrogen fertilizer use in cropland resulted in a slight decrease in GWP since the nitrogen-stimulated CO_2_ sink was slightly larger than the increased N_2_O emission, which has also been previously reported (e.g., Del Grosso et al. 2006; Zaehle et al. 2011; Tian et al. 2011a, 2012c). Our previous studies (i.e., Tian et al. 2011a, 2012b) also implied that the nitrogen fertilizer-induced CO_2_ sink might be overturned by increasing CH_4_ and N_2_O sources if present-level or more fertilizer is applied in the near future. Through its effects on restraining plant growth, elevated tropospheric O_3_ concentration cumulatively increased GWP by 3.93 Pg CO_2_ eq (0.13 Pg CO_2_ eq/year) during the entire period, which is close to the contribution of climate change. Elevated O_3_ concentration resulted in large emissions of CO_2_ to the atmosphere though it slightly decreased N_2_O and CH_4_ emissions (Xu et al. 2012).

It is notable that interactive effects among multiple environmental factors resulted in a large net reduction in GWP of 4.07 Pg CO_2_ eq during the study period. This effect was close to that from climate change and tropospheric O_3_ pollution, implying that the interactive effect among multiple environmental factors is a significant effect and should not be neglected. Recently, some field experiments with a few manipulated environmental factors were conducted to examine GHG responses at several sites, such as the SPRUCE experiment site (http://mnspruce.ornl.gov/; experiment: warming + increased atmospheric CO_2_; target: CO_2_ and CH_4_ fluxes), the Duke Forest FACE site (Experiment: CO_2_ + nitrogen fertilization; target: CO_2_ and CH_4_ fluxes), and the Aspen FACE Experiment (Experiment: CO_2_ + ozone; target: CO_2_ flux). Future studies should have more field evidence to test model representation of interactive environmental effects.

Although the major sources for terrestrial CH_4_ (i.e., wetlands) and N_2_O (i.e., cropland) have been slightly shrinking in North America over the past century (Dahl 1990; CCSP 2007; EPA 2011), the changes of other environmental factors such as climate, O_3_ pollution, and nitrogen fertilizer uses may still increase GWP. For example, large-scale drought events occurred in 2002 that have reduced the carbon sink by 20 % in the US alone (Xiao et al. 2011), on the other hand, CH_4_ and N_2_O emissions have not been greatly changed, resulting in a positive GWP in this year (Fig. 1). As estimated (USGCRP 2009), the US average temperature has increased more than 2 °F over the past 50 years and is projected to increase more in the future; the magnitude of resulting GWP change primarily depends on the amount of emitted heat-trapping gases (e.g., CH_4_, CO_2_ and N_2_O) and the sensitivity of climate to these emissions. Our study indicated that the decreased CO_2_ sink magnified the GWP increase caused by CH_4_ and N_2_O emissions under extreme climate events, suggesting GWP could be greatly increased due to more frequent climate extremes across North America in the future.

### Spatial characteristics of GWP in North America

**Fig. 3:**
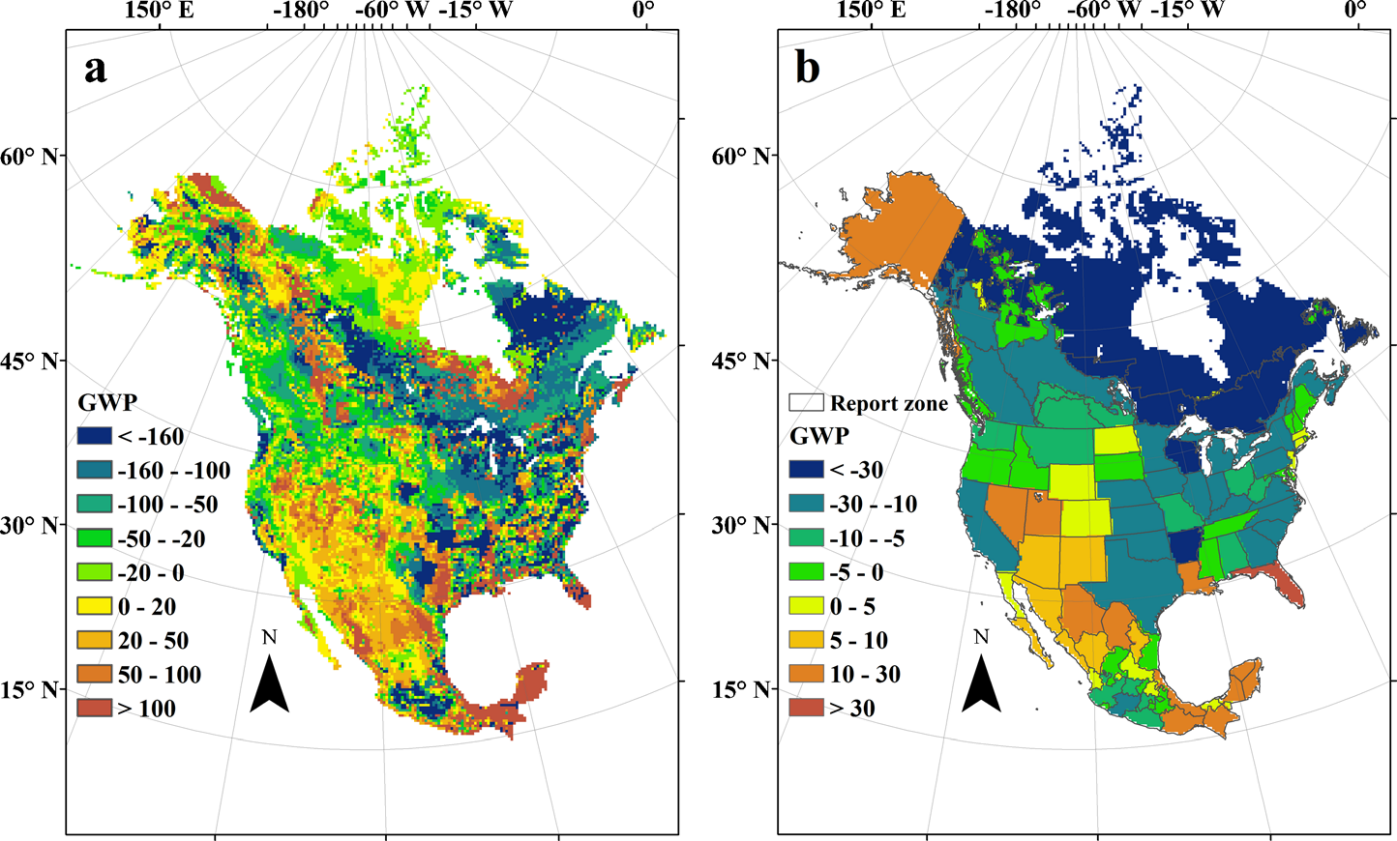
Spatial patterns of combined global warming potential for CO_2_, CH_4_ and N_2_O fluxes in North American terrestrial ecosystems during 2001–2010 (**a**: pixel level, unit: g CO_2_ eq/m^2^/year; **b**: reporting zones (delineated as Hayes et al. 2012, unit: Tg CO_2_ eq/year/zone)

The spatial and temporal variations of environmental factors (i.e., nitrogen deposition, atmospheric CO_2_, land use and land cover, cropland management, and climate) led to large spatial heterogeneities of CO_2_, CH_4_, N_2_O fluxes and their overall GWP (Fig. 3). The overall GWP was generally negative in most areas of the eastern portion of North America, while positive values concentrated in the western and southern portions. The spatial distribution pattern of the overall GWP was more consistent with that of the CO_2_ fluxes than CH_4_ and N_2_O (Fig. 3a). The highest positive GWP (>100 g CO_2_ eq/m^2^/year) was generally located in wetland areas due to higher CH_4_ emissions and in the tropical forests of the eastern Mexico due to the higher CO_2_ and N_2_O emissions (Tian et al. 2010; Xu et al. 2012). The lowest negative GWP (<−160 g CO_2_ eq/m^2^/year) occurred in the southeastern and northeastern portions of North America. To further clarify the spatial patterns, we divided the study region as countries, reporting zones, and biome types.

There are large variations among the three countries in terms of their contributions to GWP. The US had the highest capability to reduce GWP (−0.53 ± 0.38 Pg CO_2_ eq/year) though a wider estimate range was obtained as compared to Canada and Mexico (Table 1). The US has acted as the highest N_2_O source and CO_2_ sink compared to the other two countries. GWP in Mexico is positive due to higher N_2_O emission than the terrestrial CO_2_ and CH_4_ uptake, where N_2_O and CH_4_ fluxes offset about 329 % ± 119 % of the CO_2_ sink. A small negative GWP (−0.09 ± 0.21 Pg CO_2_ eq/year) was found in Canada, due to a relatively high CO_2_ sink and low N_2_O emission, which has also been reported in previous studies, such as Chen et al. (2000), Kurz and Apps (1999), Tian et al. (2012b) and Xu et al. (2012). We further divided the three countries into 97 reporting zones based on the boundaries shown in Hayes et al. (2012). At reporting zone level, we found a positive GWP in Alaska, Nevada, Florida, Louisiana and most states in Mexico, indicating these zones were potential contributors to global warming (Fig. 3b); most zones in Canada and in the northern and central US were characterized by a negative GWP. The larger emissions of CH_4_ from wetlands and N_2_O fluxes from subtropical forests had offset the large CO_2_ sinks in Louisiana and Florida. The larger N_2_O emissions and smaller CO_2_ sinks in subtropical and tropical Mexico make most states in this country a positive GWP.

All biomes except wetland had negative GWP due to environmental changes, indicating a contribution to slow down global warming in these biomes (Table 2). Among them, forest (−0.48 Pg CO_2_ eq/year) was the largest CO_2_ and CH_4_ sink but it did not mitigate climate warming as much as we expected due to higher N_2_O emissions than other natural biome types. Wetland was a relatively large CO_2_ sink; however, CH_4_ emissions exceeded the CO_2_ sink, resulting in a positive GWP of 0.37 Pg CO_2_ eq/year. Due to intensive management practices such as irrigation and nitrogen fertilizer use, cropland became a very important CO_2_ sink over recent decades (Hayes et al. 2012), although it only covers about 11 % of the total land area of North America. Despite having the highest N_2_O emissions, the overall GWP of three gases in cropland was still negative and only second to forest, contributing to a reduction of climate warming under current environmental conditions. Grassland played a nearly neutral role to the overall GWP changes in North America. Other biome types (e.g., shrubland, urban lawn, and tundra) also had a negative GWP.

**Table 2:** CO_2_, CH_4_ and N_2_O fluxes and their overall global warming potential (Pg CO_2_ eq/year) for major biome types in North America during the recent decade

Variables	Forest^a^	Grassland	Wetland	Cropland	Others^b^	North America
CO_2_	−0.69	−0.12	−0.31	−0.46	−0.27	−1.85
CH_4_	−0.04	−0.02	0.61	0.01	−0.04	0.52
N_2_O	0.25	0.11	0.07	0.23	0.15	0.81
Overall GWP	−0.48	−0.02	0.37	−0.22	−0.16	−0.50

^a^Part of forest is included in wetland as woody wetland. Biome-level GWP may not sum to the totals due to rounding

^b^Others include shrubland, tundra, desert, urban lawn and forest, and bare ground

### Uncertainty ranges of overall GWP

The DLEM simulation showed that 73 ± 14 % of the North American terrestrial CO_2_ sink was offset by CH_4_ and N_2_O emissions in the 2000s. If we include results from other continental-scale reports, the estimate spread would be wider. Given the CO_2_ sink estimates from forward modeling (i.e., 1.87 ± 1.51 Pg CO_2_ eq/year), inverse modeling (i.e., 3.41 ± 1.39 Pg CO_2_ eq/year), and inventory data (1.21 Pg CO_2_ eq/year) (Hayes et al. 2012; Huntzinger et al. 2012), the overall GWP of three GHGs was −0.53, −2.07, and 0.13 Pg CO_2_ eq/year, respectively. The CH_4_ and N_2_O emissions could offset 71 %, 39 %, and 111 % of the cooling effects from CO_2_ uptake as estimated by above approaches, respectively. It implies more research is needed to reconcile the estimated magnitude of CO_2_ balance in North American terrestrial ecosystems. If we consider the multi-source estimates of CO_2_, CH_4_ and N_2_O fluxes (Potter et al. 2006; CCSP 2007; Zhuang et al. 2007; Xiao et al. 2011; Hayes, et al. 2012; Huntzinger et al. 2012; Tian et al. 2012b), the spread of the offset ratio would be even broader (27–130 %, Supplementary Material Table S1). We found that CO_2_, CH_4_ and N_2_O fluxes ranged between −3.56– −1.17, 0.16–0.50, and 0.80–1.02 Pg CO_2_ eq/year, respectively (Fig. S3). The narrower range for N_2_O is due to less available data for comparisons. The DLEM-estimated terrestrial contribution to alleviate climate warming in terms of GWP (−0.50 ± 0.27 Pg CO_2_ eq/year) is slightly smaller than the mean value of all the existing estimates (−0.90 ± 1.33 Pg CO_2_ eq/year; Table S1).

By combining DLEM-estimated CH_4_ and N_2_O fluxes with CO_2_ flux estimates obtained from inverse modeling, forward modeling, and inventory-based estimates (Hayes et al. 2012; Huntzinger et al. 2012; King et al. 2012), we also examined the spatial variation in GWP uncertainty (Supplementary Material Fig. S3). All three methods showed a positive GWP in Alaska and some zones in the southern portion of North America, while most zones in the north-central US were characterized by negative GWP. According to the inventory-based estimate, most of the zones in Mexico, south-central US, West Pacific regions, and northern Canada had positive GWP. However, in terms of the inverse modeling results, all of the reporting zones in Canada and the US except Florida showed negative GWP. This indicated that the spatial pattern of estimated GWP still has large uncertainty. Therefore, further studies are needed to identify our knowledge gaps, diminish uncertainty ranges, and find convergence among GHG estimates from different approaches.

### Implications, limitations and research needs

Both CH_4_ and N_2_O emissions were predicted to greatly increase in the near future due to environmental changes over different continents (US EPA 2012; Zaehle et al. 2011; Schulze et al. 2009; Koven et al. 2011; Tian et al. 2012b). These predictions imply that climate warming may be accelerated by GHG emissions from North American terrestrial ecosystems, especially in the years with extreme climate events. Some management practices, such as nitrogen fertilizer use and manure application, have been reported to greatly increase carbon sequestration in cropland (CCSP 2007); however, our studies indicated these practices would also greatly increase N_2_O emission after a long-term application, especially when nitrogen saturation or fertilizer overuses occur (Tian et al. 2012a, b; Del Grosso et al. 2006). Nitrogen fertilizer has been widely used in planted forests in the US (Fox et al. 2007); however, it is still uncertain how this affects N_2_O and CH_4_ fluxes though it increases carbon storage. Therefore, a comprehensive approach should be used to evaluate the consequences of these management practices on the combined GHG balance in North America in the future.

As a process-based terrestrial ecosystem model, DLEM builds on the understanding of biogeochemical processes controlling C, N and water dynamics and can be used to simulate the dynamics of multiple ecosystem components (Tian et al. 2010). However, substantial uncertainties might exist in the ecosystem modeling results because some processes are still underrepresented (Hayes et al. 2012; Banger et al. 2012). For example, the hydrological processes in wetlands are not well-represented yet in most terrestrial ecosystem models, which could result in large uncertainties in simulating GHG fluxes, especially for CH_4_ and CO_2_ in wetlands (Riley et al. 2011; Sulman et al. 2012; Melton et al. 2013). In addition, the model driving data also vary substantially. For example, there exist several series of climate data (e.g., CRU, NCEP1, NCEP2, CRUNCEP, etc.) and land use and land cover data (Spahni et al. 2011; Huntzinger et al. 2012), which may produce different modeling results. Besides, larger uncertainties also come from differences among various approaches.

To narrow down the estimate range, we need a better understanding of critical biogeochemical processes which control land–atmosphere GHG exchanges, interactions among multiple environmental factors, and classification and distribution of key vegetation cover types, such as natural wetland and inundation extent. A synthesis of the available data from multiple sources and a framework allowing full accounting of all three GHG fluxes will be applicable and urgent for global change research. Generating consistent input data, as well as conducting data-model integration and model-model intercomparison are important ways to decrease the existing uncertainties. Based on the standard model simulation procedures and consistent input data sets, the intercomparisons will identify and quantify uncertainty sources for various estimates of GHG budgets resulting from different model representations, structures and parameterizations. For land-atmosphere CO_2_ fluxes, several model-data integration and model-model intercomparision projects have been or are being conducted for the North America, for example, the NACP and Multi-scale Synthesis and Terrestrial Model Intercomparison Project (MsTMIP) (Huntzinger et al. 2013). Unfortunately, there are no such synthesis activities for N_2_O and CH_4_ for this region. Therefore, for future research, we call for multi-constraint synthesis including inventories, field observations, inverse and forward modeling to achieve a “best estimate” of GHG balance and better understand the underlying mechanisms responsible for land-atmosphere GHG exchange in North America.

## Electronic supplementary material

Below is the link to the electronic supplementary material.

ESM 1 (DOCX 1264 kb)
