# Supplementary material for: North American terrestrial CO2 uptake largely offset by CH4 and N2O emissions: toward a full accounting of the greenhouse gas budget
Source: Clim Change. 2014 Mar 14;129(3):413–26. doi: 10.1007/s10584-014-1072-9 (PMC4439729; doi:10.1007/s10584-014-1072-9)
Supplement: Supplementary file 1 — (DOCX 1264 kb) [file 10584_2014_1072_MOESM1_ESM.docx]

Electronic Supplementary Material for:

**“North American terrestrial CO_2_ uptake largely offset by CH_4_ and N_2_O emissions: Toward a full accounting of the greenhouse gas budget”**

Hanqin Tian· Guangsheng Chen· Chaoqun Lu· Xiaofeng Xu· Daniel J. Hayes· Wei Ren· Shufen Pan· Deborah N. Huntzinger· Steven C. Wofsy

Hanqin Tian*·Guangsheng Chen· Chaoqun Lu· Xiaofeng Xu· Wei Ren· Shufen Pan

International Center for Climate and Global Change Research and School of Forestry and Wildlife Sciences, Auburn University, Auburn, AL 36849, USA

*E-mail: [tianhan@auburn.edu](mailto:tianhan@auburn.edu)

Telephone: 1-334-844-1059

Fax: 1-334-844-1084

Daniel J. Hayes· Xiaofeng Xu

Environmental Sciences Division, Oak Ridge National Laboratory, Oak Ridge, TN 37831, USA

Deborah N. Huntzinger

School of Earth Sciences and Environmental Sustainability, North Arizona University, Flagstaff, AZ 86011, USA

Steven C. Wofsy

Department of Earth and Planetary Science, Harvard University, 29 Oxford St., Cambridge, MA 02138, USA

**1. Simulations of greenhouse gas fluxes**

In this Supplementary Material, we briefly present the key processes involved in simulating land-atmosphere exchanges of CO_2_, CH_4_ and N_2_O in the Dynamic Land Ecosystem Model (DLEM).

*1.1 Simulations for CO_2_ dynamics in DLEM*

The detailed carbon cycling processes in the DLEM model has been described in Tian et al. (2010; 2011; 2012). The uptake of atmospheric CO_2_ by vegetation during photosynthesis is represented by Gross Primary productivity (*GPP*) in the DLEM. Carbon dioxide is returned to the atmosphere from the autotrophic respiration of plants (*R_A_*) and heterotrophic respiration (*R_H_*) associated with decomposition. Net primary production (*NPP*) is calculated as the difference between *GPP* and *R_A_*. The net carbon exchange of CO_2_ between the terrestrial biosphere and the atmosphere from natural ecosystem metabolism is represented by net ecosystem production (*NEP*), which is calculated as the difference between *NPP* and *R_H_*. The DLEM also accounts for the C fluxes during land conversion among different plant functional types and the sum of C emissions (*E_C_*) from decay of agricultural and wood products (*E_P_*). In addition, CH_4_ emissions (*F_CH4_)* are deducted from the C fluxes. Thus, the net C exchange (*NCE*, g C/m^2^) is calculated as follows:

*NCE* = *GPP* – *R_A_* – *R_H_* – *E_C_* – *E_P_* – *F_CH4_* = *NEP* – *E_C_* – *E_P_* – *F_CH4_*  (1)

A positive value of NCE represents a gain of C in terrestrial ecosystems whereas a negative value represents a C loss to the atmosphere.

*1.2 Simulations for CH_4_ dynamics in DLEM*

Methane flux module in the DLEM had been described in detail by Tian et al. (2010b; 2011a). DLEM simulates CH_4_ production, consumption, and transport. Due to relatively small contribution from other substrates (Conrad 1996; Mer and Roger 2001), DLEM only considers the CH_4_ production from dissolved organic carbon (DOC), which is indirectly controlled by environmental factors including soil pH, temperature and soil moisture content. The DOC was produced through three pathways: GPP allocation, and decomposition byproducts from soil organic matter and litterfall. CH_4_ oxidation, including the oxidation during CH_4_ transport to the atmosphere, CH_4_ oxidation in the soil/water, and atmospheric CH_4_ oxidation on the soil surface, is determined by CH_4_ concentrations in the air or soil/water, as well as soil moisture, pH, and temperature. Most CH_4_-related biogeochemical reactions in the DLEM were described as the Michaelis-Menten equation with two coefficients: maximum reaction rate and half-saturated coefficient. Three pathways for CH_4_ transport from soil to the atmosphere-ebullition, diffusion, and plant-mediated transport-are considered (Tian et al. 2010). It is assumed that methane-related biogeochemical processes only occur in the top 50-cm soil layer. Net CH_4_ flux between the atmosphere and soil is determined by the following equation:

$F_{CH4}=F_{P}+F_{D}+F_{E}-F_{air, oxid}-F_{trans, oxid}$ (2)

where *F_CH4_* is the net flux of CH_4_ between soil and the atmosphere (g C/m^2^/d); *F_P_* is plant-mediated transport from soil pore water to the atmosphere (g C/m^2^/d); *F_D_* is the diffusive flux of CH_4_ from water surface to the atmosphere (g C/m^2^/d); *F_E_* is the ebullitive CH_4_ emission to the atmosphere; *F_air, oxid_* is the rate of atmospheric methane oxidation (g C/m^2^/d); *F_trans, oxid_* is the oxidized CH_4_ during plant-mediated transport (g C/m^2^/d).

*1.3 Simulations for N_2_O dynamics in DLEM*

The N_2_O dynamic module in DLEM has been described in detail by Tian et al. (2010b; 2011a) and Xu et al. (2012). Major nitrogen cycling processes in the terrestrial ecosystems include nitrogen input from the atmosphere (through nitrogen deposition and nitrogen fixation), fertilizer input, nitrogen immobilization/mineralization, plant N uptake, nitrification/denitrification, adsorption/desorption, nitrogen leaching, and unknown nitrogen loss through fire or other disturbances. N_2_O emissions are primarily from the soil nitrogen transformation processes (i.e., nitrification and denitrification).

Nitrification, a process converting ammonium into nitrate, is simulated as a function of soil temperature, moisture, and the NH_4_^+^ concentration. Denitrification, through which the nitrate is converted into nitrous gases, is simulated in the DLEM as a function of soil temperature, moisture, and the NO_3_^-^ concentration. All the products of nitrification and denitrification that leave the system are N-containing gases. The empirical equation reported by Davidson et al (Davidson et al. 2000) is used to separate N_2_O from other gases (mainly NO and N_2_). The equations for calculating nitrification, denitrification and N_2_O fluxes are:

$N_{nit}=\min(N_{pot,nit}, N_{NH4})$ (3)

$N_{pot,nit}=V_{nit, max}\times f_{nit}(N_{NH4})\times f_{nit}(T_{soil})\times f_{nit}(vwc)$ (4)

$N_{denit}=\min(N_{pot,denit}, N_{NO3})$ (5)

$N_{pot,denit}=V_{denit, max}\times f_{denit}(N_{NO3})\times f_{denit}(T_{soil})\times f_{denit}(vwc)$ (6)

$F_{N2O}=(0.001\times N_{nit}+N_{denit})\times\frac{{10}^{(\frac{vwc}{\emptyset}\times0.026-1.66)}}{(1+{10}^{(\frac{vwc}{\emptyset}\times0.026-1.66)})}$ (7)

Where, $N_{nit}$ and $N_{denit}$are the nitrification and denitrification rates (g N/m^2^/d), respectively; $N_{pot,nit}$ and $N_{pot,denit}$ are the potential nitrification and denitrification rates (g N/m^2^/d), respectively; $N_{NH4}$and $N_{NO3}$are the concentrations of NH_4_^+^ and NO_3_^-^ in the soil (g N/m^2^), respectively; $V_{nit, max}$ and $V_{denit, max}$ are the potential (or maximum) nitrification and denitrification rates without limitation (g N/m^2^/d), respectively;$f_{nit}(N_{NH4})$ and $f_{denit}(N_{NO3})$ are scalars that represent the effects of soil NH_4_^+^ and NO_3_^-^concentration on nitrification rate, respectively; $f_{nit}(T_{soil})$ and $f_{denit}\left( T_{soil} \right)$ are soil temperature scalars that represent the effect of soil temperature on nitrification and denitrification, respectively; $T_{soil}$ is the soil temperature (^o^C); $f_{nit}(vwc)$ and $f_{denit}(vwc)$ are soil moisture scalars that denote the effect of water content on nitrification and denitrification, respectively; $vwc$ is the soil volumetric water content; $F_{N2O}$is the N_2_O flux from soil to the atmosphere (g N/m^2^/d); 0.001 is the proportion of nitrification product released as gaseous nitrogen (Lin et al. 2000); $\emptyset$ is the soil porosity.

**2. Model Input Data**

The climate dataset during 1979-2010 was generated based on North American Regional Reanalysis (NARR) dataset (http://nomads.ncdc.noaa.gov/data.php?name=access#narr datasets). The maximum, minimum and average temperatures were calculated based on the eight 3-h records in one day. Precipitation, solar radiation, and relative humidity were directly derived from the NARR dataset. Land-use and land-cover change data were extracted from a global data set developed by History Database of the Global Environment (HYDE 3.0). Ozone data was retrieved from the global dataset developed by Felzer et al. (2005) covering 1900–2050. Annual nitrogen deposition data were retrieved from a global data set that was extrapolated from three yearly maps (Dentener et al. 2006). Soil properties data, including soil texture, soil pH, soil bulk density, were extracted from a global data set Global Soil Data Task posted online in the Oak Ridge National Laboratory (daac.ornl.gov). Nitrogen fertilizer use data for North America was developed by combining several data sources, including Food and Agriculture Organization (FAO) country-level data (www.fao.org), US county-level data (www.usda.gov), and Canada provincial level data (www.cfi.ca). The annual atmospheric concentration of CO_2_ before 1959 was estimated by VEMAP (The Vegetation/Ecosystem Modeling and Analysis Project), and the data after 1959 were provided by National Oceanic and Atmospheric Administration (NOAA) (www.esrl.noaa.gov). The distributional map of contemporary vegetation types was developed using multiple sources of data, including global land-cover derived from Landsat imagery (De Fries et al. 1998), National Land Cover Dataset 2000 (www.usgs.gov), and global database of lakes, reservoirs and wetland (Lehner and Döll 2004). All the datasets were transformed and re-projected to a consistent projection system for driving the DLEM model.

The interannual variations of major environmental factors were shown in Fig. S2. Mean annual temperatures showed a significant increasing rate of 0.03 ± 0.01^o^C/yr, while precipitation did not show a significant change trend; however, significant higher precipitation was found during 2003-2008. Nitrogen fertilizer use, nitrogen deposition, atmospheric CO_2_ concentration and tropospheric O_3_ concentration significantly increased since 1979. The annual increasing rates were estimated to be 0.04 ppm-hr for O_3_ pollution, 0.044 Tg N/yr for nitrogen deposition, 0.12 Tg N/yr for nitrogen fertilizer use, and 1.66 ppm/yr for atmospheric CO_2_ concentration (Tian et al. 2010b). The cropland area decreased from 2.59 million km^2^ to 2.51 million km^2^, while forest, shrubland, grassland and wetland area changed in very small magnitude. Spatial variations of these input data were described in detail in Xu et al. (2010). The natural wetlands primarily distribute in Alaska, the western Canada, the Hudson Bay, the eastern US coast. The highest tropospheric O_3_ pollution, as high as 5000 ppb-hr (monthly cumulative hourly O_3_ dose over a threshold of 40ppb in ppb-hr), occurred in the northwestern and southeastern US. The cropland with the highest nitrogen fertilizer use (larger than 10 g N/m^2^/yr) was primarily located in the US. Canada and Mexico had small cropland area and low nitrogen fertilizer use. The highest nitrogen deposition was found in the southeastern US.

**3. Model parameterization and implementation**

The Bayesian method was used to determine the optimal values for key parameters related to CO_2_, CH_4_ and N_2_O processes (Robert and Casella, 2005; Ricciuto et al., 2008). The priori values for these major parameters were given first, and then based on these priori parameters and field observational CO_2_, CH_4_ and N_2_O fluxes, we tune the optimal values for those parameters. The parameter values that give the best fit to the observational fluxes were considered as the optimal parameters and used for the regional simulation. The major parameters and their values related to CH_4_ and N_2_O were listed in Tian et al. (2010) and Xu et al. (2010, 2012), and the major parameters related to CO_2_ fluxes were shown in Tian et al. (2012a), Hayes et al. (2012), and Schwalm et al. (2010).

We used the potential vegetation map, long-term mean climate during 1979–2008, the concentrations of tropospheric ozone and atmospheric CO_2_, and nitrogen deposition in 1900 to feed the DLEM model to approach an equilibrium state (i.e., the inter-annual variations are < 0.1 g C/m^2^ for carbon storage, and < 0.1 g N/m^2^ for nitrogen storage). After the system reached equilibrium state for potential vegetation, we ran the model again for another 500 years to an equilibrium state for cropland and urban areas. Then we randomly select climate data from 1979 to 2008 to spin-up for 10 times (totally 300 years for spin-up run). Other model input data were kept constant at the 1900 level during spin-up run. Finally, the model was run in a transient mode with input data from 1901 to 2010. The annual climate data between 1901 and 1978 were developed by randomly assigning a year between 1979 and 2010. Only the outputs between 1979 and 2010 were analyzed to show the spatial and temporal patterns of CO_2_, CH_4_ and N_2_O fluxes in the North American terrestrial ecosystems.

**4. Uncertainty ranges through synthesizing all existing estimates**

Through synthesizing all existing regional estimates for terrestrial CO_2_, CH_4_ and N_2_O fluxes in North America, we found a large uncertainty range for combined GWP, which was primarily due to the larger uncertainty of CO_2_ fluxes (Table S1). The narrower uncertainty ranges for CH_4_ and N_2_O were because of fewer estimates available for synthesis (Tian et al. 2012b).

At the continental scale, CO_2_ flux data obtained from inverse modeling, forward modeling, and inventory-based estimate (Hayes et al. 2012; Huntzinger et al. 2012; King et al. 2012) were compiled to explore the spatial uncertainty of the GWP (Fig. S3). The sources and methods for inventory- and modeling based CO_2_ fluxes were described in detail by Hayes et al. (2012) and Huntzinger et al. (2012). For the contemporary analysis for CO_2_ fluxes, 97 reporting zones were divided (Hayes et al. 2012). These zones cover the majority of US states, Canadian managed ecoregions, and Mexican states for which inventory data were available. Estimates of CH_4_ and N_2_O fluxes were solely derived from DLEM simulation since no other spatially-explicit result is available. The combined GWP for three gases were analyzed for these 97 reporting zones.

**References**

Chen G, Tian H, Zhang C et al (2012) Drought in the Southern United States over the 20^th^ century: Variability and its impacts on terrestrial ecosystem productivity and carbon storage. Climatic Change DOI 10.1007/s10584-012-0410-z

De Fries R, Hansen M, Townshend J, Sohlberg R (1998) Global land cover classiﬁcations at 8 km spatial resolution: the use of training data derived from Landsat imagery in decision tree classiﬁers. Int J Remote Sens 19:3141–3168

Dentener F (2006) Global maps of atmospheric nitrogen deposition, 1860, 1993, and 2050, Dataset, available at: <http://daac.ornl.gov/> from Oak Ridge National Laboratory Distributed Active Archive Center, Oak Ridge, Tennessee, USA

Felzer B, Reilly J, Melillo J, et al (2005) Future effects of ozone on carbon sequestration and climate change policy using a global biogeochemical model. Climatic Change 73:345–373, doi:10.1007/s10584-005-6776-4

Hayes DJ, Turner DP, Stinson G, et al (2012) Reconciling estimates of the contemporary North American carbon balance among terrestrial biosphere models, atmospheric inversions, and a new approach for estimating net ecosystem exchange from inventory-based data. Glob Change Biol 18: 1282–1299

Huntzinger DN, Post WM, Wei Y, et al (2012) North American Carbon Program (NACP) regional interim synthesis: Terrestrial biospheric model intercomparison. Ecol Model 232:144-157

Lehner B, Doll P (2004) Development and validation of a global ¨database of lakes, reservoirs and wetlands. J Hydrol 296: 1–22

Lin BH, Sakoda A, Shibasaki R, Goto N, Suzuki M (2000) Modeling a global biogeochemical nitrogen cycle in terrestrial ecosystems. Ecol Model 135:89–110

Liu M, Tian HQ, Chen GS, et al (2008) Effects of land use and land cover change on evapotranspiration and water yield in China during the 20^th^ century. J Am Water Resour Assoc (JAWRA) 44:1193-1207:

Liu, M., H. Tian, C. Lu, X. Xu, G. Chen, W. Ren. 2012. Effects of Multiple Environment Stresses on Evapotranspiration and Runoff over the Eastern China. J Hydrol [426–427](http://www.sciencedirect.com/science/journal/00221694/426): 39–54.

Lu C, Tian H, Liu M et al (2012) Effect of nitrogen deposition on China’s terrestrial carbon uptake in the context of multi-factor environmental changes. Ecol Appl 22:53–75

Lu C, Tian H (2013) Net greenhouse gas balance in response to nitrogen enrichment: Perspectives from a coupled biogeochemical model. Glob Change Biol 19:571-588. doi: 10.1111/gcb.12049

Mer JL, Roger P (2001) Production, oxidation, emission and consumption of methane by soils: a review. Eur J Soil Biol 37: 25–50

Ren W, Tian HQ, Liu M, et al (2007) Tropospheric ozone pollution and its influence on net primary productivity and carbon storage in terrestrial ecosystems of China. Journal of Geophysical Research, 112, D22S09, doi: 10.1029/2007JD008521.

Ren W, Tian H, Xu X et al (2011) Spatial and temporal patterns of CO_2_ and CH_4_ fluxes in China’s croplands in response to multifactor environmental changes. Tellus B doi: 10.1111/j.1600-0889.2010.00522.x

Ren W, Tian H, Tao B, Huang Y, Pan Y (2012a) China’s crop productivity and soil carbon storage as influenced by multifactor global change. Gobl Change Biol doi: 10.1111/j.1365-2486.2012.02741.x

Ren W, Tian H, Tao B, et al (2012b) Impacts of ozone pollution and climate change on net primary productivity and carbon storage of China’s forest ecosystems as assessed by using a process-based ecosystem model. Glob Ecol Biogeogr doi: 10.1111/j.1466-8283.2010.00606.x

Schwalm CR, Williams CA, Schaefer K et al (2010) A model-data intercomparison of CO_2_ exchange across North America: Results from the North American Carbon Program Site Synthesis. J Geophy Res doi:10.1029/2009JG001229

Tao B, Tian H, Chen G, et al (2013) Terrestrial carbon balance in tropical Asia: contribution from cropland expansion and land management. Global and Planetary Change <http://dx.doi.org/10.1016/j.gloplacha.2012.09.006>.

Tian HQ, Chen G, Liu M, et al (2010a) Model Estimates of Ecosystem Net Primary Productivity, Evapotranspiration, and Water Use Efficiency in the Southern United States during 1895-2007. For Ecol and Manage 259:1311–1327.

Tian H, Xu X, Liu M et al (2010b) Spatial and temporal patterns of CH_4_ and N_2_O fluxes in terrestrial ecosystems of North America during 1979–2008: application of a global biogeochemistry model. Biogeosciences 7:2673–2694

Tian H, Xu X, Lu C et al (2011a) Net exchanges of CO_2_, CH_4_, and N_2_O between China’s terrestrial ecosystems and the atmosphere and their contributions to global climate warming. J Geophy Res, G02011, doi: 10.1029/2010JG001393

Tian H, Melillo J, Lu C et al (2011b) China’s terrestrial carbon balance: Contribution of multiple global change factors. Global Biogeochemical Cycles, doi:10.1029/2010GB003838.

**Tian H, Lu C, Chen G et al (2011c) Climate and land use controls over terrestrial water use efficiency in monsoon Asia. Ecohydrol 4:322–340.**

Tian H, Chen G, Zhang C et al (2012a) Century-scale responses of ecosystem carbon storage and flux to multiple environmental changes in the southern United States. Ecosystems 15:674–694

Tian H, Lu C, Chen G et al (2012b) Contemporary and projected biogenic fluxes of methane and nitrous oxide in terrestrial ecosystems of North America, Front Ecol Environ 10:528–536

Wang L, Tian H, Song C, Xu X, Chen G, Ren Wand Lu C. (2012) Net exchanges of CO_2_, CH_4_ and N_2_O between marshland and the atmosphere in Northeast China as influenced by multiple global environmental changes. Atmos Environ 63:77–85

Xu X, Tian H, Zhang C, Liu M, Ren W, Chen G, Lu C, Bruhwiler L (2010) Attribution of spatial and temporal variations in terrestrial methane flux over North America. Biogeosciences 7: 3637–3655

Xu X, Tian H, Liu M, Ren W, Chen G, Lu C, Zhang C (2012a) Multiple-factor controls on terrestrial N_2_O flux over North America. Biogeosciences 9:1351–1366

Xu X, Tian H (2012b) Methane exchange between marshland and the atmosphere over China during 1949-2008. Glob Biogeochem Cy, doi:10.1029/2010GB003946

Zhang C, Tian H, Chappelka A, Ren W, Chen H, Pan S, Liu M, Styers D, Chen G and Wang Y. (2007) Impacts of climatic and atmospheric changes on carbon dynamics in the Great Smoky Mountain National Park. Environ Pollut 149: 336–347.

Zhang C Tian H Wang Y Zeng T Liu Y (2010) Predicting response of fuel load to future changes in climate and atmospheric composition in the Southern United States. For Ecol Manage, doi:10.1016/j.foreco.2010.05.012.

Zhang C, Tian H, Chen G et al (2012) Impacts of urbanization on carbon balance in terrestrial ecosystems of the Southern United States. Environ Pollut 164:89–101

**Tables and Figures:**

**Table S1** Uncertainty ranges for CO_2_, CH_4_ and N_2_O fluxes and their overall global warming potential (Pg CO_2_ eq/yr) in the 2000s through synthesizing all existing estimates

| Countries | CO_2_* | CH_4_ | N_2_O | Overall GWP | Offset rate by CH_4_ and N_2_O (%) |
| --- | --- | --- | --- | --- | --- |
| US | -2.57~-0.73 | 0.095~0.27 | 0.48~0.69 | -1.99~0.23 | 22~131 |
| Canada | -0.88~-0.44 | 0.083~0.24 | 0.14~0.15 | -0.66~-0.05 | 25~89 |
| Mexico | -0.037~-0.11 | -0.019~-0.01 | 0.18 | 0.052~0.15 | 147~515 |
| North America | -3.56~-1.17  (-2.16 ± 1.13) | 0.16~0.50  (0.35 ± 0.14) | 0.80~1.02  (0.91 ± 0.06) | -2.60~0.35  (-0.90 ± 1.33) | 27~130  (58 ± 28) |

*Data synthesis for CH_4_ and N_2_O fluxes in the North American terrestrial ecosystems were summarized in Tian et al. (2012); Data sources for terrestrial CO_2_ fluxes: Huntzinger et al. 2012; CCSP 2007; Deng et al. 2007; Xiao et al. 2011; Pacala et al. 2001; Ju et al. 2006; Hayes et al. 2012. The data in parenthesis indicate the mean value ± 2 standard errors (95% confidence interval).


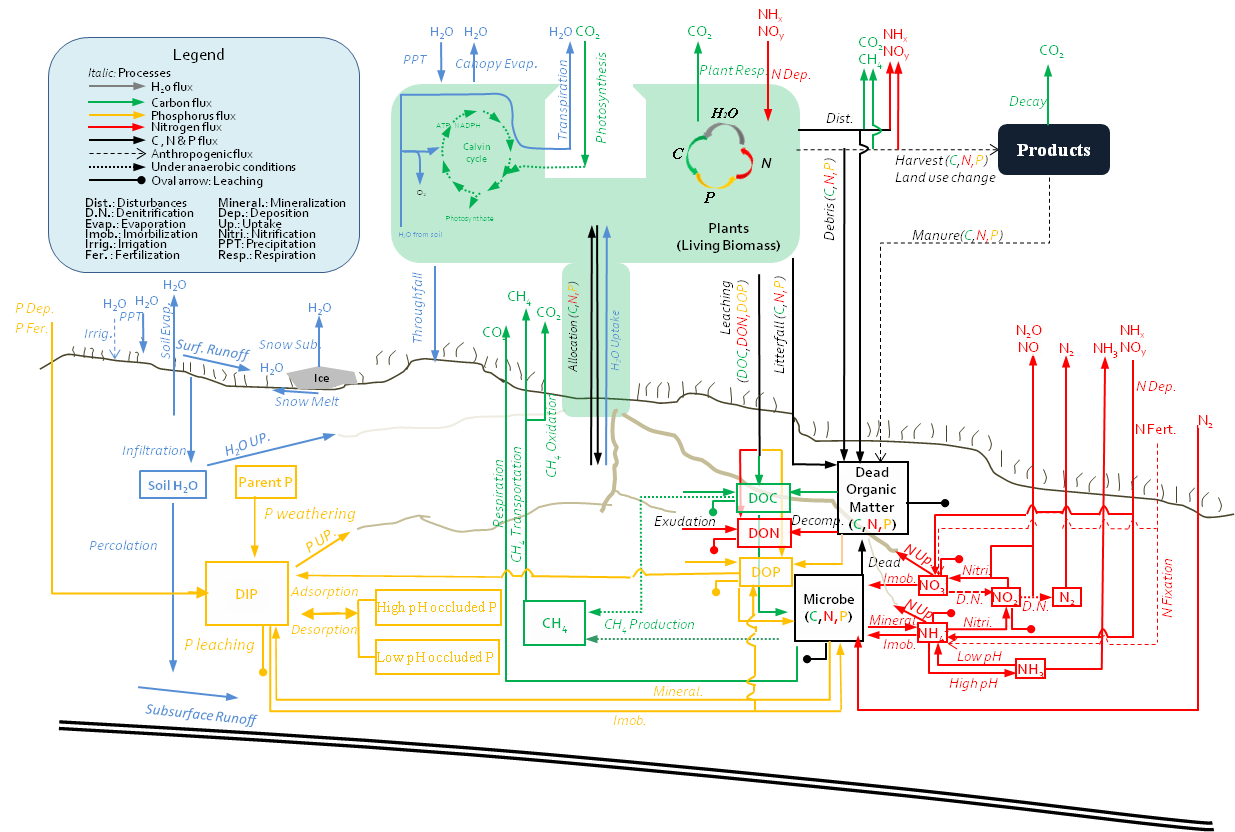


**Fig. S1 Schematic illustration of the key biogeochemical and hydrological processes as represented by the Dynamic Land Ecosystem Model (DLEM)**


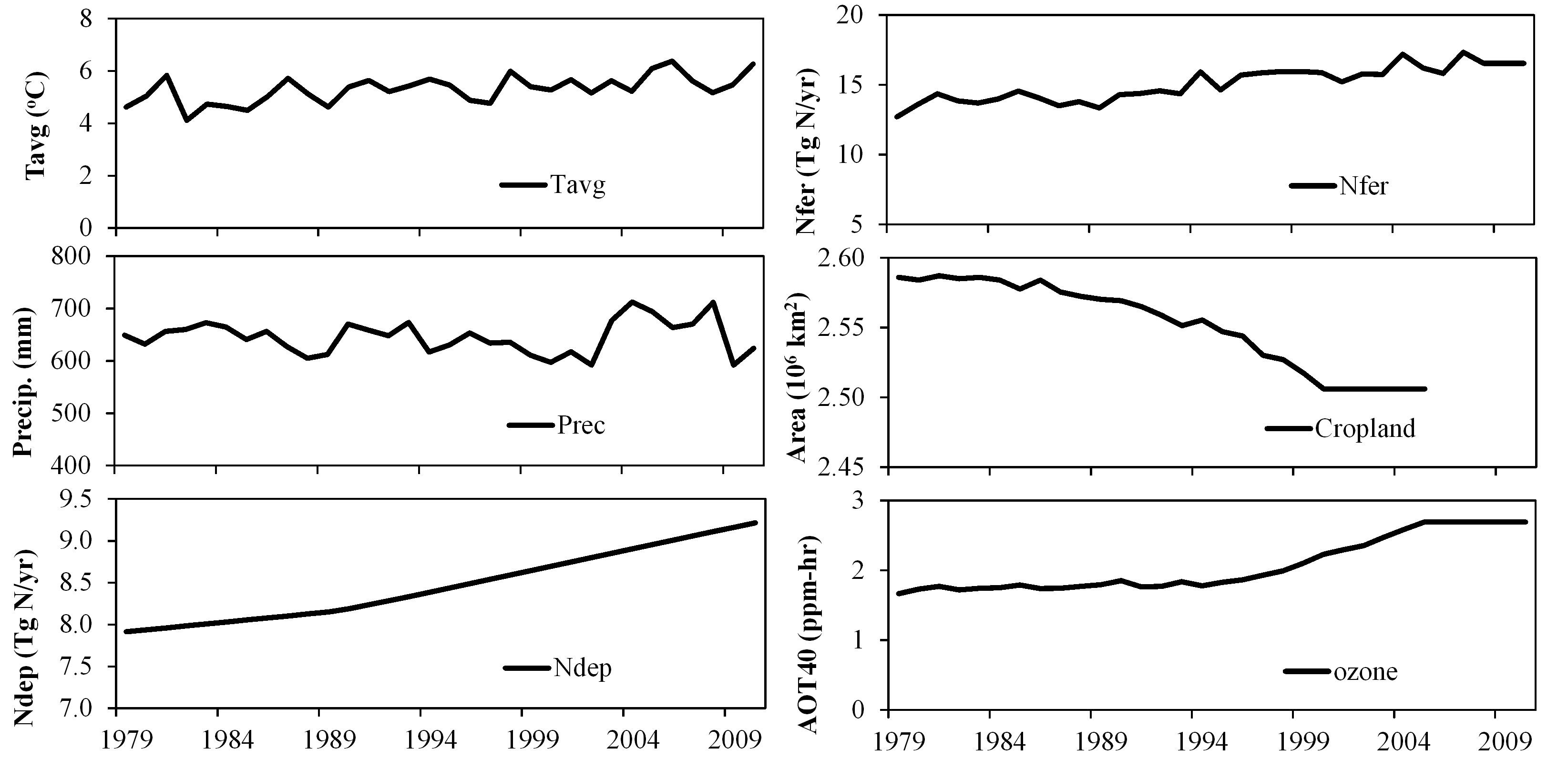


**c**

**d**

**f**

**e**

**b**

**a**

**Fig. S2** Interannual variations of major environmental factors in North America. Note: mean temperature (a, ^o^C), total nitrogen fertilizer use amount (b, Tg N/yr), total precipitation (c, mm), cropland area (d, 10^6^ km^2^), nitrogen deposition (e, Tg N/yr), and tropospheric ozone pollution (f, ppm-hr; AOT40: monthly cumulative hourly O_3_ dose over a threshold of 40 ppb-hr)


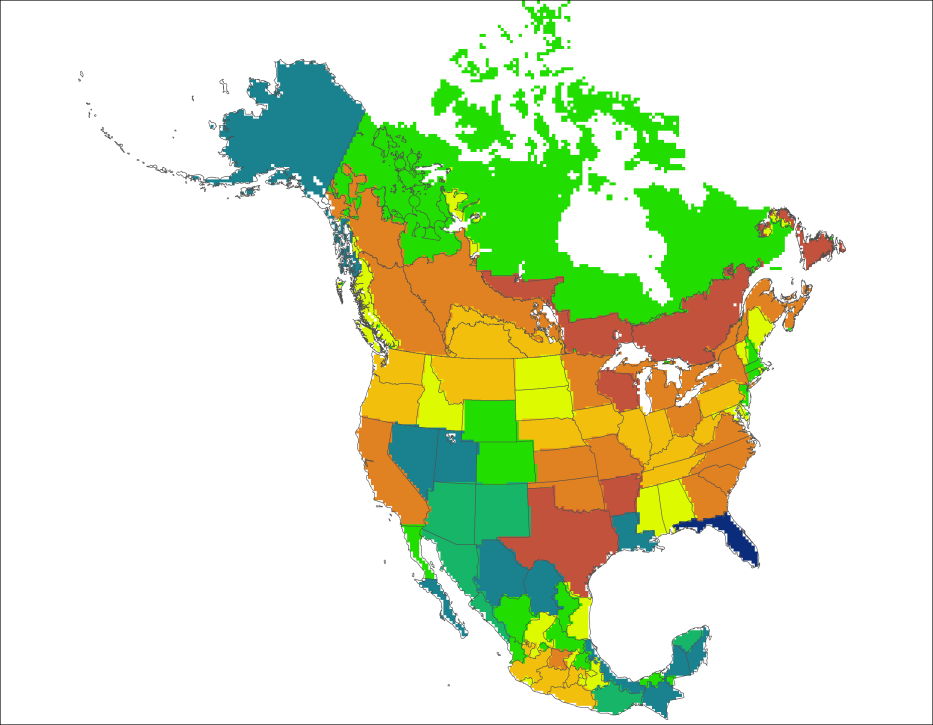

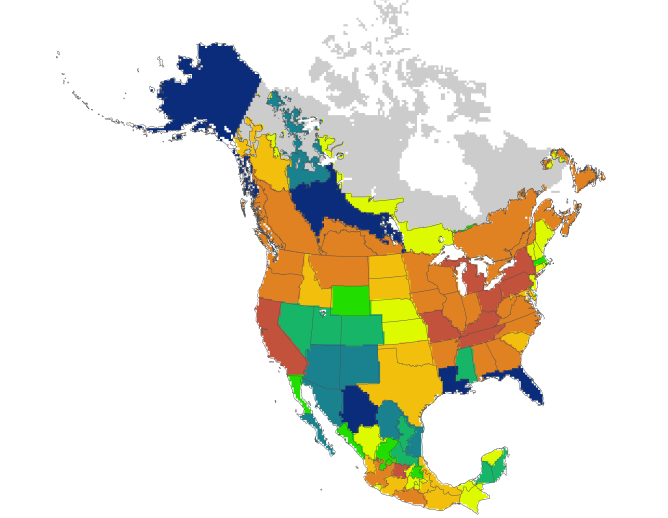

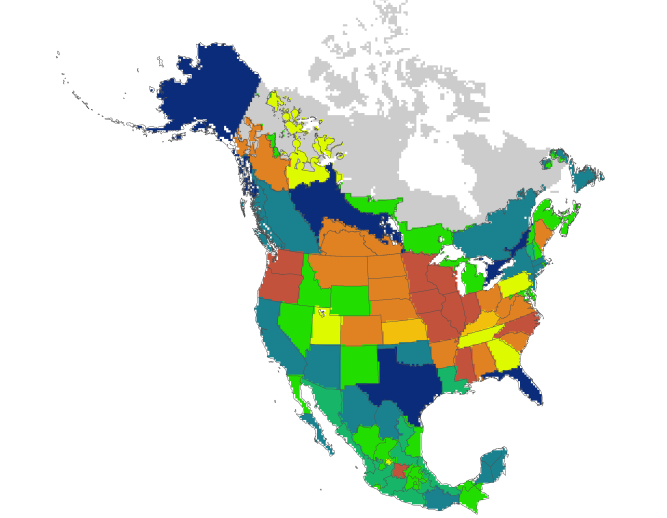

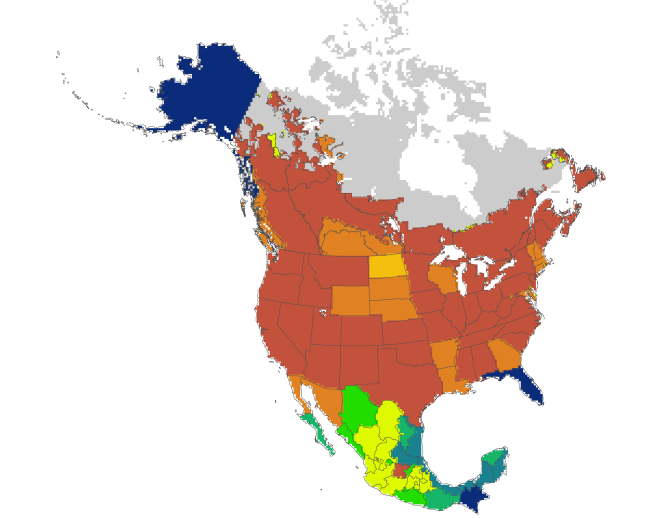


**d**

**b**

**c**

**a**

**Fig. S3** Global warming potential (Tg CO_2_ eq/yr) estimated by combining DLEM-simulated CH4 and N2O fluxes with CO2 estimates from the inventories (a), inverse models (b), forward models (c), and DLEM model alone (d) for 97 reporting zones in North America during recent decade
